# Color Tunable, Lithography-Free Refractory Metal–Oxide Metacoatings with a Graded Refractive Index Profile

**DOI:** 10.1021/acs.nanolett.2c04867

**Published:** 2023-03-30

**Authors:** Joshua Perkins, Haoyang Cheng, Chris Craig, Daniel W. Hewak, Behrad Gholipour

**Affiliations:** † Nanoscale Optics Lab, Department of Electrical and Computer Engineering, University of Alberta, T6G 2R3, Edmonton, Canada; ‡ Optoelectronics Research Centre (ORC), University of Southampton, Southampton SO17 1BJ, U.K.

**Keywords:** Metamaterials, metal oxides, graded index, metacoating, structural color

## Abstract

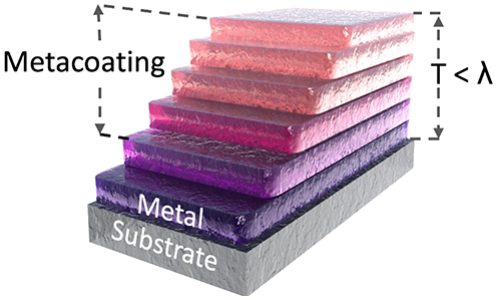

The refractory metal–oxide semiconductors are an overlooked platform for nanophononics that offer alloys with high melting points and tunable optical constants through stoichiometry changes and ion intercalation. We show that these semiconductors can form metamaterial coatings (metacoatings) made from a set of highly subwavelength, periodic metal–oxide layers (≤20 nm) with a varying and graded refractive index profile that includes a combination of high and low refractive indices and plasmonic layers. These metacoatings exhibit vibrant, structural color arising from the periodic index profile that can be tuned across the visible spectrum, over ultralarge lateral areas through bottom-up thermal annealing techniques.

The unprecedented functionalities and electromagnetic properties engineered using the metamaterials and metasurface paradigm in various nanophotonic device platforms throughout the past decade arise from subwavelength periodic and/or nonperiodic metamolecule resonators embedded within a host dielectric or plasmonic medium of a particular chemical composition. Precise micro-/nanostructuring of the geometry of the resonators enables the realization of remarkable electromagnetic behavior due to the engineered effective permittivity and permeability, and by extension the refractive index. Photonic metamaterials research has traditionally focused on noble plasmonic metals which afford no compositional tunability, relatively low-melting-points, high optical losses, and a lack of CMOS compatibility. To combat these losses and instabilities, researchers are actively exploring alternative material platforms to plasmonic metals such as chalcogenide semiconductors, transparent conductive oxides, nitrides, and 2D materials.^1−3^ While refractory metal-nitrides have garnered much attention, a versatile and stoichiometrically tunable material platform formed by the refractory metal–oxides has been somewhat overlooked.^4^ The refectory metal–oxide material family offers a vast compositional space filled with binary and ternary dielectric alloys that offer a wide range of refractive indices and extinction coefficients that can be tuned by additional oxidation, ion-species incorporation, or by gas exposure. Notably, alloys such as WO_3_, Fe_2_O_3_, CuO, Cu_2_O, and CeO_2_, in particular have higher dielectric resonator figures of merit than those offered by popular dielectrics widely used in nanophotonics such as GST, BST, VO_2_, GaP, GZO, and ITO across the visible wavelength range.^5^

Refractory metal–oxides can be directly deposited through physical vapor deposition techniques or grown from a seed metallic layer by oxidation. The oxidation of metals is a thermal process that can occur through direct annealing or laser-induced oxidation of metallic films.^6,7^ In both cases, thermal oxidation starts with the impingement of oxygen molecules (O_2_) onto a heated metallic film. The molecular oxygen is physiosorbed onto the surface and dissociated into oxygen (O) atoms that are then chemically bonded to metal atoms (M) at the film’s surface.^6,8^ The bonded oxygen and metal atoms reorientate their chemical bond into the lowest energy state by exchanging the internal M atom with the external O atom driving the O atom into the metal film. This process, known as “place exchange”, leads to the growth of the oxide film one monolayer at a time. The thermal oxidation proceeds by the transportation of oxygen and metal ions through the metal–oxide monolayers to the metal or air interface, where additional oxidation reactions can occur, leading to an increase in oxide film thickness with time.^9−11^ Therefore, as film thickness increases the additional oxide volume does not strictly form with the same concentration of O or M atoms across the thickness of the film. Depending on the M and O species used, this can result in the formation of multiple discrete oxide layers with graded stoichiometric profiles, due to the nonlinear diffusion profiles of M and O ions across the thickness of the film.^6,9,11^ The resulting concentration gradients formed across the thickness can lead to the formation of films with graded refractive index profiles that can be precisely tuned through the thermal oxidation process. The oxidation process, if left to continue, eventually homogenizes all monolayers through the oxide film until the metallic film is fully oxidized, the film temperature drops below the oxidation temperature of the metal, or the oxygen gas is removed or depleted from the oxidation environment. Therefore, during the thermal oxidation of metals, below a homogenization threshold, the varying diffusion of metal, metal oxide, and oxygen species can lead to a stoichiometric grading across the thickness of the oxide which translates to graded optical properties across the nanoscale thickness of the grown metal–oxide film.^12−15^ The resulting optical properties arise from the interplay of the graded refractive index profile of all the layers and can act as the basis for a myriad of optoelectronic functionalities.

Thin films having graded elemental content have found use in data storage, protective coatings, and as optical materials. Electrical contacts in the form of W plugs can be thermally annealed to form a graded WO_*x*_ coating in resistive RAM (RRAM) technologies to achieve high endurance and thermal stability in nonvolatile memory devices.^16^ Additionally Hf_*x*_Al_1–x_O_*y*_ films with a graded Al profile have been shown to exhibit multilevel electronic switching capabilities that can be used to form an artificial synapse for neuromorphic computing applications.^17^ Most notably, graded transparent conductive oxide films have also been explored as antireflective optical coatings or GRIN materials based on graded cosputtering of ITO or TiO_2_ with SiO_2_ to form multilayer stacks.^18,19^ Though the exploration of graded films is typically emulated by modulated deposition routines few researchers have found thermal annealing of parent materials (Sn, In, and In+Sn) can be performed to form SnO_2_, In_2_O_3_, and ITO which exhibit an optical response that can best be described when employing graded optical constants.^20,21^ Furthermore, films with controlled, graded optical constant have been shown to hold great promise for even wider nanophotonic applications and can be used as graded index lenses in super-resolution microscopy, and can provide better resolution in photolithography techniques.^22−24^

Here we show that precisely tuned inhomogeneous thermal oxidation can be used as a cheap, high-accuracy fabrication method for the realization of large lateral area metamaterial coatings (metacoatings), we demonstrate the bottom-up growth of a graded tungsten oxide (WO_*x*_) coating with subwavelength overall thickness, and highly subwavelength interlayers that form a large lateral area color-tunable coating on a seed tungsten (W) surface. In our experiments, W films are deposited through sputtering and used as seed layers for thermal oxidation at elevated temperatures (up to >400°C) in ambient atmospheres (Figure 1a, see also methods). Post thermal oxidation, below the homogenization threshold, a mixed phase tungsten oxide film is obtained as observed through X-ray diffraction (XRD) measurements (Figure 1b).

**Figure 1:**
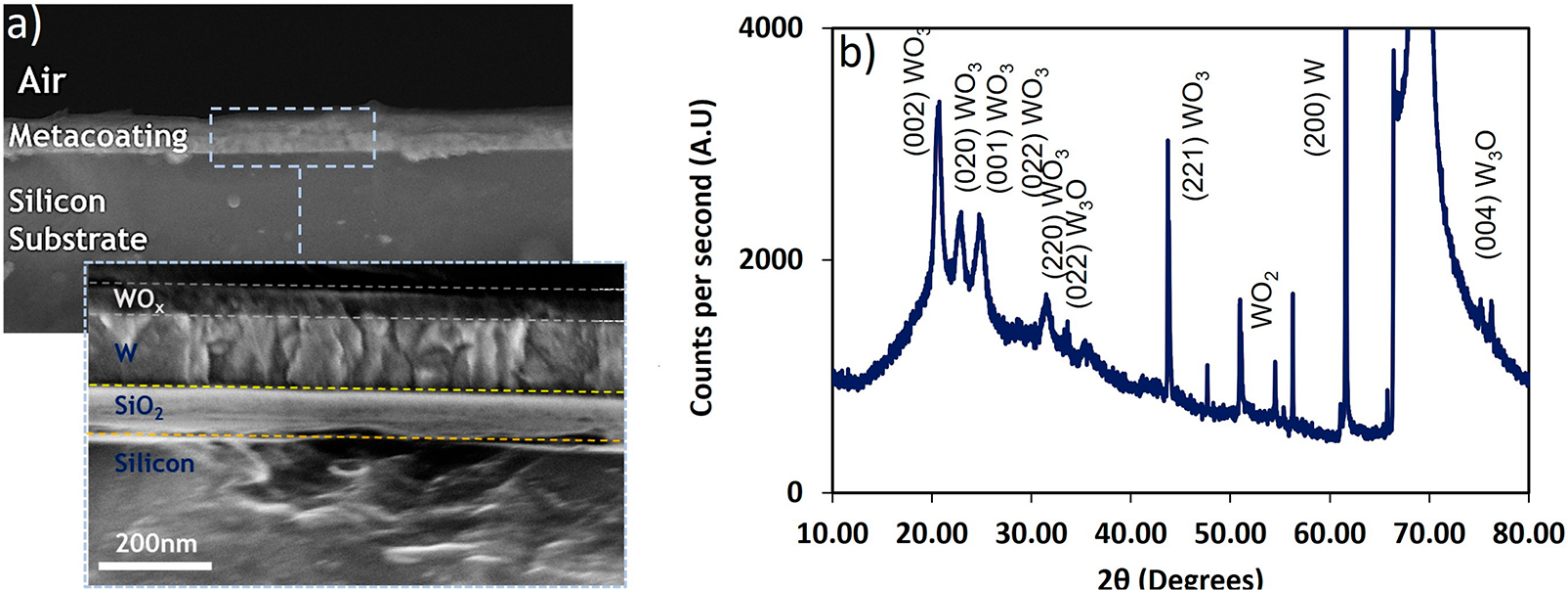
Material characteristics of a tungsten oxide graded index metacoating. (a) Scanning electron microscope image of the cross-section of a grown metacoating on a silicon substrate showing the tungsten oxide (WO_*x*_), tungsten (W), native oxide (SiO_2_) and Si substrate layers. (b) Xray diffraction (XRD) spectrum of a metacoating on Si showing multiple diffraction peaks corresponding to WO_3_, WO_2_, W, and W_3_O stoichiometries.^25−27^

Optically thick tungsten is an absorbing medium across ultraviolet/visible wavelengths with a dispersion of reflectivity that is relatively constant. This flat dispersion can be modified through precise oxidation of the metallic film to yield a range of large-area, chip-scale vibrant structural colors (Figure 2a,b). By adjusting the oxidation time, and through that the thickness, *T*, of the oxide layer a wide color gamut can be obtained (Figure 2c). As mentioned, research in this direction has often focused on costly physical vapor deposition (PVD) of multilayers (e.g hyperbolic metamaterial devices) or lithography/focused ion-beam enabled lateral (nano)structures such as conventional plasmonic or dielectric metasurfaces in which color is realized via out-of-plane structural design at the subwavelength scale.^28,29^ Here, we show that as opposed to conventional metamaterials and metasurfaces, metal–oxide metacoatings enable the realization of a tunable optical response across large lateral areas without the need for any lithography, based on an in-plane, highly subwavelength graded refractive index profile that can be engineered through controlled thermal oxidation of seed metallic films.

**Figure 2:**
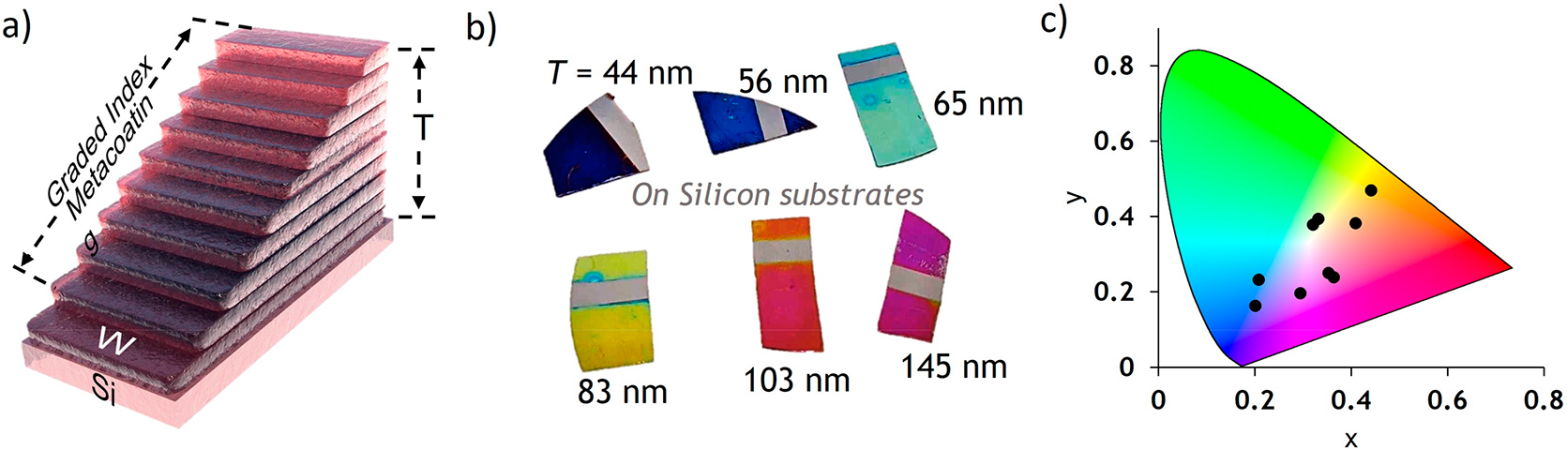
Structural color characteristics of graded index tungsten oxide metacoatings. (a) An illustration of a metacoating showing the graded index layers on Si substrates. (b) Metacoatings grown in ambient conditions at 410 °C on a laboratory grade hot plate to form metacoatings. Metacoatings have thickness from 44 to 145 nm demonstrating vibrant color from purple (*T* = 44 nm) to red (*T* = 145 nm) with intermediate colors of blue, green, yellow and orange. (c) The fabricated metacoatings color gamut in the corresponding CIE 1931 color space coordinates showing a range of vibrant colors.

The unpolarized optical response of the metacoatings as measured using microspectrophotometery and shown in Figure 3a demonstrates the emergence of a broad dip in reflection upon oxidation that enables filtering any desired band of the UV/Visible spectrum. This is realized through the emergence of a subwavelength-thickness oxide film on the reflective metallic tungsten surface and the precise control of its thickness by changing annealing/oxidation times, giving rise to the range of observed structural colors. As with any metamaterial system the optical response of the metacoatings can be tuned across various spectral bands through the adjustment of subwavelength structural components within the system. Figure 3b highlights the lithography-free structural tunability of the optical response that is possible in such metacoatings, showing a shift from λ = 400 nm to λ = 542 nm resulting from a corresponding film thickness increase of 100 nm obtained through careful adjustment of annealing/oxidation times.

**Figure 3:**
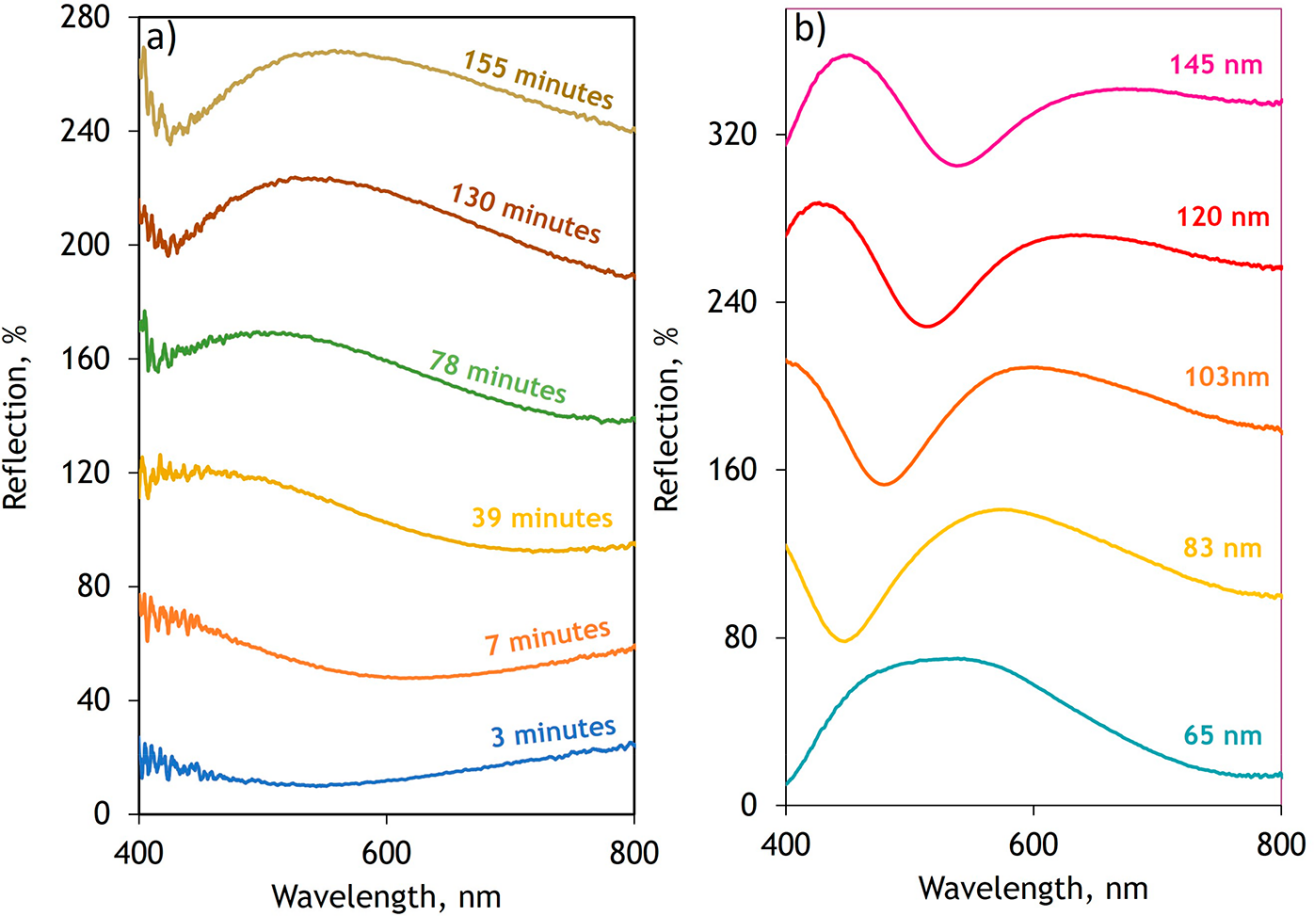
Dynamic and static spectrophotometry of graded oxide metacoatings. a) Reflection spectra of a metacoating dynamically measured over time, during annealing in a confined pressured controlled CDA atmosphere. Sample is grown from a 60 nm thick W seed layer. b) Static reflection measurements of metacoatings with various thicknesses grown from a 200 nm thick W seed layer on a laboratory hotplate.

The layered internal structure of a metacoating is best probed using variable angle spectroscopic ellipsometry that enables the further elucidation of the optical response of each subwavelength oxide layer. The results confirm the dependence of the observed optical properties on the highly subwavelength graded refractive index profile and its interplay with the metallic backplane.

The dispersion of refractive index and extinction coefficient of the individual layers for the case of a *T* = 103 nm film across UV to near-infrared wavelengths (400 nm < λ < 800 nm) is shown in Figure 4a,b. This reveals high refractive index (*n* > 3) upper layers close to the metal–oxide air interface and low-index, plasmonic buried layers that are comparable to vacuum (*n* ≥ 1) at the metal—metal–oxide interface (see Figure S2 for permittivity). The number of layers that best describes the optical performance of the metacoatings increases with overall thickness showing an increase from four to seven, of approximately *L* = 20 nm thick layers, when increasing the overall film thickness from *T* = 83 to 134 nm.

**Figure 4:**
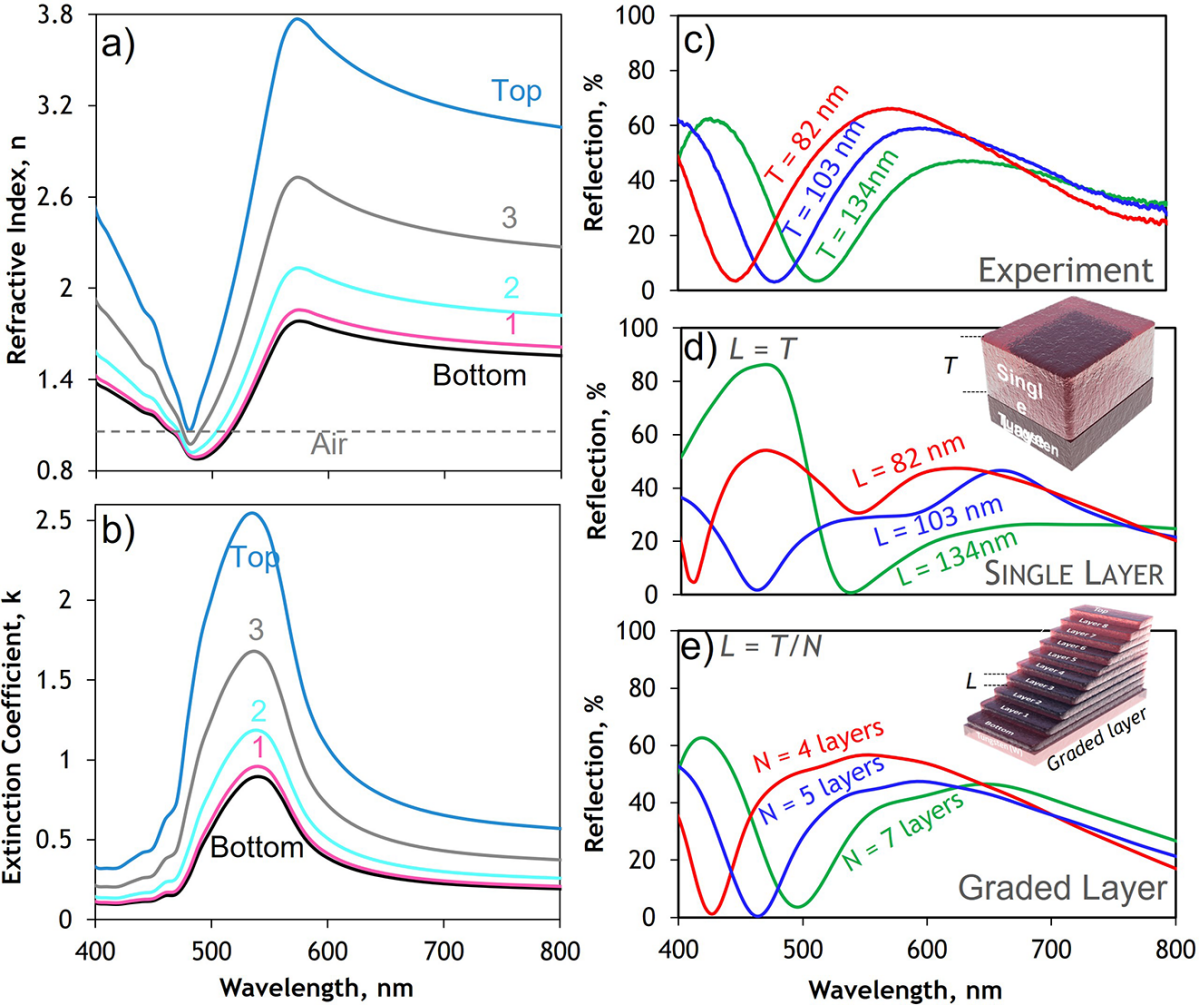
Optical properties of subwavelength metacoating interlayers. a) Extracted refractive index (*n*) profile of the graded index metacoating modeled with *N* = 5 layers where high indices at the air-film (Top layer) interface and low indices (Bottom layer) at the oxide-metal interface b) Extracted extinction coefficient (k) profile showing high k at the Top layer and low k towards the bottom layers c) Experimentally measured reflection spectrum for three metacoating thicknesses (*T* = 82 nm, 103 nm and 134nm) (d) FDTD simulated film reflection spectra for metacoatings modeled with a homogenous single layer approach where layer thickness, *L*, is the total of the individual metacoating and (e) a multilayered graded refractive index profile with *N*= 4, 5, and 7 discreet oxide layers with layer thicknesses of *L* ≈ 20 nm where *T* = 82 nm, 103 nm and 134 nm respectively. Optical constants for the fitted permittivities of the metacoating are presented in Figure S2.

To further confirm this, finite-difference time-domain (FDTD) simulations (Figure 4d,e), are performed using the experimentally measured optical constants shown in Figure 4a,b. The model assumes a semi-infinite silicon substrate, normal incident narrowband plane-wave illumination and, due to the periodic boundary conditions, a metacoating of infinite extent along the substrate surface. The simulated response shows a very poor agreement when assuming that the film is a singular layer (Figure 4d). However, when a multilayered, graded index profile is considered, there is very good qualitative and quantitative agreement between the experimentally measured (Figure 4c) and numerically simulated (Figures 4e) reflection spectra (also see Figure S1 for the simulated angle dependence of the response).

Inhomogeneous thermal oxidation is key to the growth and tunable performance shown in these metacoatings. This is further reaffirmed by depositing a 300 nm thick WO_3_ film through the reactive RF sputtering PVD technique. Variable angle spectroscopic ellipsometry measurements and subsequent FDTD simulations reveal that, in contrast to the thermally oxidized metacoatings, as this film has been grown through a homogeneous vapor deposition method, it does not possess and rely upon a multilayered graded index profile as the basis for its observed optical response. Here, a single layer best describes the observed response (Figure 5).

**Figure 5:**
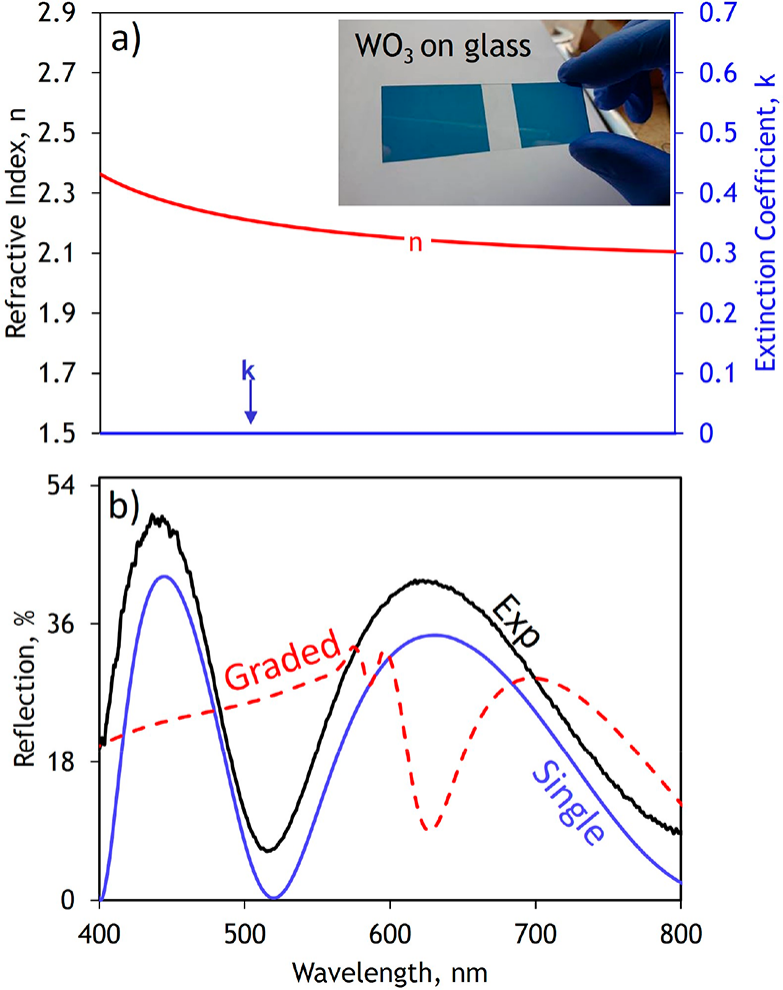
Optical Dispersion data and Experimental (Exp) and Simulated reflection response a) Optical dispersion data for *T* = 300 nm PVD deposited, homogenous, WO_3_ films modeled with a homogenous refractive index/extinction coefficient profile measured using variable angle spectroscopic ellipsometry B) Experimental (Exp) and simulated reflection spectra of the *T* = 300 nm PVD film using both graded approach (where *N* = 5 layers) and the homogenous optical dispersion shown in (a).

In conclusion, we have shown that thermal annealing techniques can be used for the bottom-up growth of large-area metacoatings made from refractory metal–oxides. As opposed to coloring achieved by multilayer depositions or traditional planar metasurfaces, the vibrant coloring observed from the demonstrated metacoating is completely determined by interlayer structural design and can be made independent of the polarization. Through carefully tailoring the thermal cycling temperature–time profile through a hot plate or pulsed-laser heating, arbitrary oxidation profiles can be created, further expanding the available color gamut. Refractory metal–oxides provide a robust and promising platform for automotive and aerospace functional coatings and in emerging adaptive and reconfigurable environmental monitoring and computing devices.

In particular, the metal–oxide metacoatings presented in this work can be grown on a variety of surfaces from wafer-scale to large industrial-scale parts and components, using localized laser oxidation or by large area thermal oxidation through the use of high-capacity furnaces. Based on the chosen oxidation temperature, tailored oxidation times can be achieved (Figure S3). Furthermore, metal–oxides are a widely used platform in electronic memristive technologies where movement of oxygen atoms within a host lattice provides the means for changes in conductivity and refractive index that can be exploited in subsequent reconfigurable metacoating device platforms. Therefore, their use within nanophotonic metamaterial and metacoatings will open the door to reconfigurable and adaptive devices enabled by the intercalation and movement of metal and gas ions within the metal–oxides.^30^ Metal–oxide materials are also sensitive to the chemistry of their surrounding environment and have been widely used in environmental monitoring and optoelectronic gas sensing platforms, which can be exploited in future flat optical sensing devices using metacoating technology.^5^

## Supporting Information Available

Experimental methods regarding fabrication, variable angle ellipsometry, static spectrophotometry, and dynamic annealing measurements, incident angle (0°, 5°, 10°, and 15°) dependent FDTD simulations of the *T* = 82 nm thickness metamaterial with *N* = 4 (Figure S1), real and imaginary components of the dielectric function corresponding to the refractive index and extinction coefficients of the metacoating in Figure 4 (Figure S2), and, additionally, the variation of annealing temperatures (380 °C, 410 °C, and 440 °C) to show (Figure S3) that the metacoatings can be fabricated at shorter or longer durations (PDF)

## Abbreviations

XRD: X-ray diffraction
CMOS: complementary metal–oxide semiconductor
RRAM: resistive random access memory
FDTD: finite difference time domain
CDA: compressed dry air
PVD: physical vapor deposition
GZO: gallium zinc oxide
GST: germanium antimony telluride
BST: bismuth antimony telluride
M: metal
O: oxide
MOx: metal oxide
