# Supplementary material for: Color Tunable, Lithography-Free Refractory Metal–Oxide Metacoatings with a Graded Refractive Index Profile
Source: Nano Lett. 2023 Mar 30;23(7):2601–6. doi: 10.1021/acs.nanolett.2c04867 (PMC10103291; doi:10.1021/acs.nanolett.2c04867)
Supplement: Supplementary file 1 — nl2c04867_si_001.pdf [file nl2c04867_si_001.pdf]

ASSOCIATED CONTENT

Supporting Information

Colour tunable, lithography-free refractory metal-oxide metacoatings with a graded refractive index profile

Joshua Perkins[1], Haoyang Cheng[1], Chris Craig[2], Daniel W. Hewak[2] and Behrad Gholipour[1]

[1] Nanoscale Optics Lab, Department of Electrical and Computer Engineering, University of Alberta, T6G 2R3, Edmonton, Canada

[2] Optoelectronics Research Centre (ORC), University of Southampton, Southampton, United Kingdom

Methods

Fabrication:

Graded-oxide metacoatings: W thin films are fabricated by cleaning Si wafer chips and glass microscopy slides with Acetone/IPA/DI water to remove organic contaminants. Following cleaning, Si chips are secured to a rotating substrate plate with Kapton tape inside a Kurt J. Lesker CMS-18 multiple planar magnetron sputtering system loaded with a 3 inch W target having 99.99% purity. The system is then pumped for 1 hour to reach a $5x10^{-6}$ Torr base pressure. Ar is introduced to bring the system to $7x10^{-3}$ Torr. A DC plasma is ignited using a MDX500 plasma source set to 300W. Following a 5-minute target burn, W is deposited onto the sample surface for 21 minutes to obtain 200nm thick W. The 200nm W films on Si are then

annealed on a laboratory hot plate in open air to obtain samples with varying thickness/optical response/color.

PVD control film: 300nm WO_3 films are deposited using a RF magnetron system (Kurt J Lesker Nano-38) with reactive gas flow. The system is pumped to 5 x 10^{-6} Torr before deposition. Ar/O_2 gasses are introduced into the chamber to achieve a 30% O_2 concentration in the chamber with a deposition pressure of 4 x 10^{-3} Torr. WO_3 films are deposited on both Si and glass.

Optical Measurements:

Spectrophotometry: Measurements are taken in the visible spectrum using an Ocean Optics HR4000 spectrometer attached to a trinocular port on a modified optical microscope from AmScope (ME580TA-PZ-2L-18M3-3PL). The spectrometer is coupled to a fiber optic multimode patch cable using F = 40mm plano convex lens at the output of the trinocular port. The system is then brought into focus on a first surface protected silver optical mirror (PF10-03-P01) from Thorlabs using an Ealing Beck 15x Reflecting Objective (0.28NA). The mirrors optical response is then measured using the spectrometer and is used as a reference spectrum for subsequent measurements. Each annealed sample on Si is then focused and measured.

For dynamic annealing measurements the microscopy chamber's lid is introduced and secured during the reference measurement to capture the effects of the ZnSe optical window. The lid is then opened, mirror removed, and samples placed on the heating stage at room temperature.

Dynamic annealing measurements: Samples are annealed in an environmentally controlled microscopy chamber (Microptik MHCS-600P) with house-supplied clean dry air (CDA). The chamber is first evacuated with an oil filled vacuum pump (Edwards ED660), and brought to 760 Torr by using a mass flow controller (MKS 1179C) and pressured transducer (MKS 980P) controlled in a PID scheme in LabVIEW software. The process gas, clean-dry-air (CDA), brings the system to a stabilized pressure before the samples are heated from ambient temperatures to 410 ˚C (measured at the surface of the heater) at a rate of 167.88 ˚C/min and held at 410 ˚C for the desired time. During heating samples are constantly illuminated with white light and measured using the same spectroscopy system detailed previously. Reflection spectra, temperature, pressure, and gas flow measurements are taken at intervals of 10 seconds for the duration of the experiment.

Variable angle spectroscopy ellipsometry:

Variable angle spectroscopic ellipsometry (VASE) is a variety of spectroscopic ellipsometry (SE) that utilizes linearly polarized light at various incident angles to measure the polarization change of reflected light due to interaction with bulk and thin films. This is accomplished by illuminating a sample with linearly polarized light and measuring the elliptically polarized light reflected from the sample. The measured light contains a relative amplitude, Ψ, and phase difference Δ, known as ellipsometry parameters. The ellipsometric parameters are able to describe the change in polarization due to material-dependent optical properties, material thickness, multiple layers or graded materials. [1,2]

We performed VASE using a WVASE system from JA Woollam. The scans are performed across 300nm – 1700nm wavelengths at 60˚, 65˚, and 70˚ incidence and experimentally measured thickness of the total metacoating thickness. The W seed layer is is measured on a

glass reference sample. The data for the W parent layer is then fit as a B-Spline layer on a 7059 Cauchy substrate with 3mm thickness. The optical model for W is extracted and used as the underlying layer in fitting the optical dispersion data of the graded oxide samples and used in subsequent FDTD simulations. The as-grown oxides are then fit using a B-spline with a nonlinear grading using Maxwell-Garnett effective media approximation.

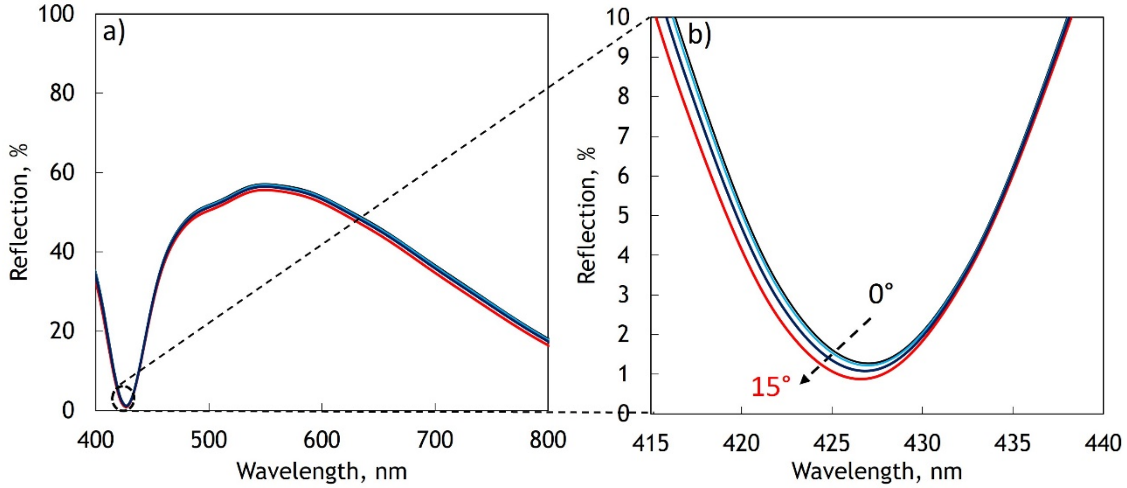

Figure S1. Incident angle dependent finite difference time to domain simulations of the T = 82nm thickness metamaterial with N = 4 layers. a) Overlayed simulated optical spectra showing overlap of optical spectra simulated with 0°, 5°, 10°, and 15° incident angles. b) A zoom in of the optical spectra's resonance illustrating the difference in optical spectra of the metacoating at 0°, 5°, 10°, and 15° incident angles. b) A zoom in of the optical spectra's resonance illustrating the difference in optical spectra of the metacoating at 0°, 5°, 10°, and 15° incident angles.

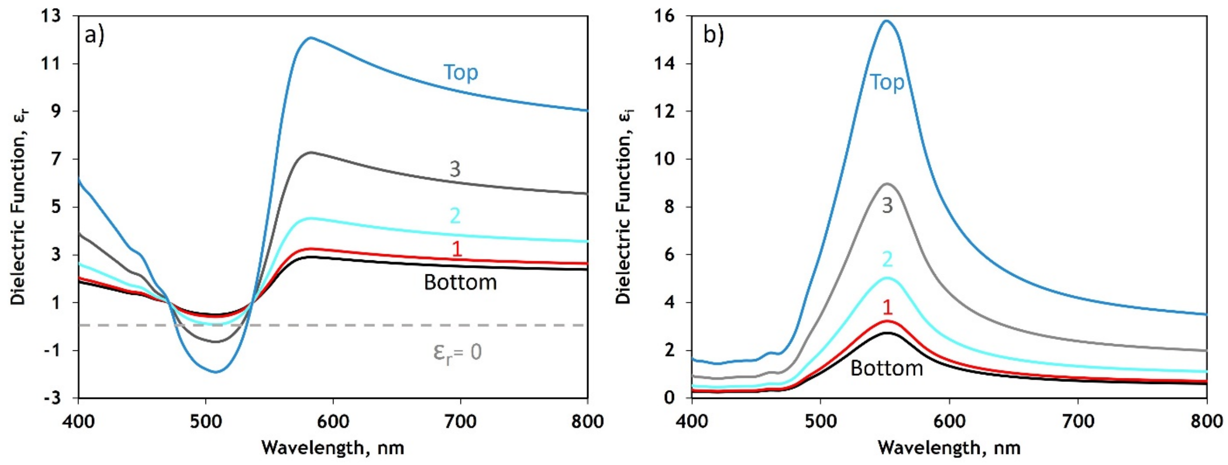

Figure S2. Optical properties of subwavelength metacoating interlayers accompanying Figure 4. a) Real portion of the dielectric function where $\varepsilon_r \approx 0$ and $\varepsilon_r < 0$ for the 1st and Bottom layers when $480\ nm \leq \lambda \leq 530 nm$. b) Imaginary portion of the dielectric function with low loss layers at the parent metal and graded oxide interface.

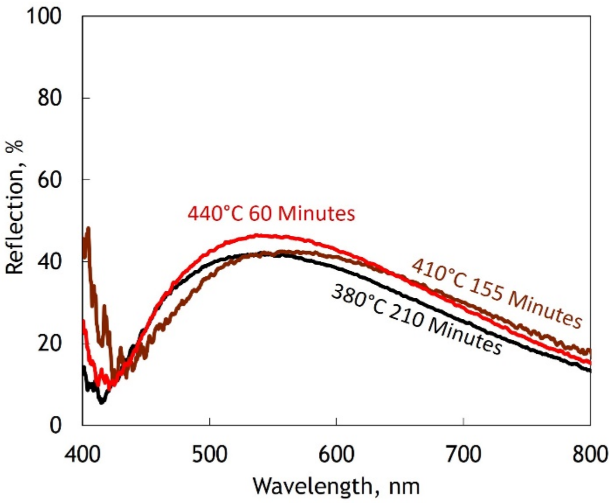

Figure S3. Spectrophotometry measurements of metacoatings annealed at 380°C, 410°C, 440 °C for 210 minutes, 155 minutes, and 60 minutes showing that the same metacoating response can be obtained by growing at lower or higher temperatures by adjusting annealing time.

References

(1). Vedam, K., Spectroscopic ellipsometry: a historical overview. *Thin solid films* **1998,** *313*, 1-9. DOI: 10.1016/S0040-6090(97)00762-1

(2). Woollam, J. A.; Snyder, P. G.; Rost, M. C., Variable angle spectroscopic ellipsometry: a non-destructive characterization technique for ultrathin and multilayer materials. *Thin Solid Films* **1988,** *166*, 317-323. DOI: 10.1016/0040-6090(88)90393-8
